# Body composition, physical capacity, and immuno-metabolic profile in community-acquired pneumonia caused by COVID-19, influenza, and bacteria: a prospective cohort study

**DOI:** 10.1038/s41366-021-01057-0

**Published:** 2022-01-05

**Authors:** Camilla Koch Ryrsø, Arnold Matovu Dungu, Maria Hein Hegelund, Andreas Vestergaard Jensen, Adin Sejdic, Daniel Faurholt-Jepsen, Rikke Krogh-Madsen, Birgitte Lindegaard

**Affiliations:** 1 Department of Pulmonary and Infectious Diseases, Nordsjællands Hospital, University of Copenhagen, Copenhagen, Denmark; 2 Centre for Physical Activity Research, Rigshospitalet, University of Copenhagen, Copenhagen, Denmark; 3 Department of Infectious Diseases, Copenhagen University Hospital, Rigshospitalet, Copenhagen, Denmark; 4 Department of Infectious Diseases, Copenhagen University Hospital, Hvidovre, Copenhagen Denmark

**Keywords:** Obesity, Pre-diabetes

## Abstract

**Background:**

Different pathogens can cause community-acquired pneumonia (CAP); however, the novel severe acute respiratory syndrome coronavirus 2 (SARS-CoV-2) causing coronavirus disease 2019 (COVID-19) has re-emphasized the vital role of respiratory viruses as a cause of CAP. The aim was to explore differences in metabolic profile, body composition, physical capacity, and inflammation between patients hospitalized with CAP caused by different etiology.

**Methods:**

A prospective study of Danish patients hospitalized with CAP caused by SARS-CoV-2, influenza, or bacteria. Fat (FM) and fat-free mass (FFM) were assessed with bioelectrical impedance analysis. Physical activity and capacity were assessed using questionnaires and handgrip strength. Plasma (p)-glucose, p-lipids, hemoglobin A1c (HbA1c), p-adiponectin, and cytokines were measured.

**Results:**

Among 164 patients with CAP, etiology did not affect admission levels of glucose, HbA1c, adiponectin, or lipids. Overall, 15.2% had known diabetes, 6.1% had undiagnosed diabetes, 51.3% had pre-diabetes, 81% had hyperglycemia, and 60% had low HDL-cholesterol, with no difference between groups. Body mass index, FM, and FFM were similar between groups, with 73% of the patients being characterized with abdominal obesity, although waist circumference was lower in patients with COVID-19. Physical capacity was similar between groups. More than 80% had low handgrip strength and low physical activity levels. Compared to patients with influenza, patients with COVID-19 had increased levels of interferon (IFN)-γ (mean difference (MD) 4.14; 95% CI 1.36–12.58; *p* = 0.008), interleukin (IL)-4 (MD 1.82; 95% CI 1.12–2.97; *p* = 0.012), IL-5 (MD 2.22; 95% CI 1.09–4.52; *p* = 0.024), and IL-6 (MD 2.41; 95% CI 1.02–5.68; *p* = 0.044) and increased IFN-γ (MD 6.10; 95% CI 2.53–14.71; *p* < 0.001) and IL-10 (MD 2.68; 95% CI 1.53–4.69; *p* < 0.001) compared to patients with bacterial CAP, but no difference in IL-1β, tumor necrosis factor-α, IL-8, IL-18, IL-12p70, C-reactive protein, and adiponectin.

**Conclusion:**

Despite higher inflammatory response in patients with COVID-19, metabolic profile, body composition, and physical capacity were similar to patients with influenza and bacterial CAP.

## Introduction

Community-acquired pneumonia (CAP) is the leading cause of hospitalization and death from infectious diseases [1]. Various pathogens cause CAP but is dominated by a range of bacteria and viruses [2]. The emergence of the novel coronavirus (severe acute respiratory syndrome coronavirus 2 [SARS-CoV-2]) causing coronavirus disease 2019 (COVID-19) has re-emphasized the vital role of respiratory viruses as a cause of CAP. Numerous studies have now identified age, male sex, and especially obesity, diabetes, hypertension, and cardiovascular disease as strong risk factors for severe COVID-19 [3]. Similar risk factors have been associated with severe outcomes from CAP caused by bacteria or influenza viruses [4, 5]. In general, people with obesity have an increased risk of admission to the intensive care unit (ICU) and mechanical ventilation [6, 7]. As a consequences, people with obesity (body mass index [BMI ]> 30) may experience diminished ventilatory drive and capacity also known as the obesity hypoventilation syndrome (Pickwickian syndrome) [8]. Obesity is associated with physical inactivity, dyslipidemia, diabetes, and cardiovascular diseases. Common for all disorders is insulin resistance, chronic low-grade inflammation, and low level of adiponectin [9]. Chronic inflammation is harmful to the immune system due to chronic leukocyte activation reducing the amount of signaling proteins in leukocytes available to respond to new stimuli [10]. People with obesity have an increased susceptibility to severe bacterial and viral infections, probably due to an impaired immune response [11, 12]. Dysregulated and/or exaggerated cytokine responses to the infection may cause immunopathology [7, 13], which is well-documented in patients with COVID-19, where several studies have described abnormal levels of cytokines [7, 14, 15]. Although patients with CAP from SARS-CoV-2 seem to have more severe outcomes than CAP caused by influenza or bacteria [5, 16], no studies have compared the degree of obesity, physical capacity, dysregulation of glucose, lipid metabolism, and inflammatory response between CAP etiologies. The aim of the study was to compare body composition, physical capacity, glucometabolic, and inflammatory profile in patients with CAP caused by SARS-CoV-2 to CAP caused by influenza virus and bacteria.

## Methods

### Study design, setting, and population

This prospective cohort study includes admission data and prospective follow-up on admission to the ICU, length of hospital stay, and mortality. The patients were prospectively recruited at Nordsjællands Hospital in Denmark from January 8, 2019, until May 26, 2020. Inclusion criteria were age ≥18 years, a new infiltrate on chest X-ray/CT, and minimum one of the following symptoms: fever (≥38.0 °C), cough, pleuritic chest pain, dyspnea, or focal chest signs on auscultation. Patients with no pathogen detection in blood, airways, or urine (*Legionella pneumophila* antigen or *Streptococcus pneumoniae* antigen) were excluded.

### Variables

#### Data collection

Information on demographic characteristics, comorbidities, symptoms, and prior medical history were collected from medical records. CURB-65 was used as a severity score [17], and participants were risk-stratified according to their CURB-65 score: mild (0–1), moderate (2), or severe (3–5). The combined burden of comorbidities was assessed by the Charlson comorbidity index (CCI) and by categorizing patients into three groups based on the sum of conditions (none, one, or more than one). In addition, microbiological test results, ICU admission, length of hospital stay, in-hospital mortality, 30-day mortality, and 180-day mortality were recorded.

#### Sampling and analysis of blood

Venous blood samples were collected within 48 h of admission and included basic blood work such as complete blood count, C-reactive protein (CRP), coagulation factors, procalcitonin, kidney function, liver function, diabetes biomarkers, and lipid status.

For analysis of cytokines, blood samples were drawn in EDTA tubes and kept on ice until centrifuged at 3000 *g* for 15 min at 4 °C. Plasma was separated from the cells and stored at −80 °C until analysis by human pro-inflammatory kits (V-PLEX Viral Panel 3 Human Kit, Meso Scale Discovery). This involved concentration of interleukin (IL)-5, interferon (IFN)-γ, IL-1β, IL-4, IL-6, IL-8, IL-10, IL-12p70, and tumor necrosis factor (TNF)-α. The concentration of IL-18 was measured by IL-18 immunoassay (U-PLEX Human IL-18 Assay, Meso Scale Discovery). The concentration of adiponectin was measured by adiponectin immunoassays (Human Adiponectin Kit, Meso Scale Discovery). All cytokines were performed in duplicates with the average presented.

#### Definition of known diabetes, undiagnosed diabetes, pre-diabetes, and hyperglycemia

Known diabetes was based on a diagnosis from patient files and/or the use of antidiabetic medicine. In patients without known diabetes, undiagnosed diabetes was defined as admission hemoglobin A1c (HbA1c) ≥48 mmol/mol (≥6.5%), and pre-diabetes was defined as admission HbA1c between 39–47 mmol/mol (5.7–6.4%) [18]. In addition, we defined normoglycemia, mild hyperglycemia, and severe hyperglycemia at admission as random blood glucose concentrations of <5.99 mmol/L, 6.0–10.99 mmol/L, and ≥11.0 mmol/L, respectively [18].

#### Study questionnaire

Study questionnaires were answered in cooperation with the patient within the first 48 h of admission. Self-reported physical activity data were recorded using the International Physical Activity Questionnaire Short Form [19]. Patients were divided into two levels of physical activity (low or moderate-to-high physical activity level). Health-related quality of life was assessed with the 5-level EQ-5D version (EQ-5D-5L) [20], while the ability to perform daily activities was evaluated with the Barthel Index-100 [21]. Frailty was assessed with the FRAIL scale [22], whereas nutritional status was assessed with the Nutritional Risk Screening-2002 [23].

#### Anthropometry and body composition

Weight was measured to the nearest 0.1 kg on an electronic scale (Seca, Hamburg, Germany), whereas height was self-reported. BMI was calculated as weight (kg)/height (m)^2^. Overweight and obesity were defined as BMI ≥ 25 kg/m^2^ and BMI ≥ 30 kg/m^2^, respectively. Waist circumference was measured to the nearest 0.5 cm. The distribution of fat mass (FM) and fat-free mass (FFM) were assessed with bioelectrical impedance analysis (BioScan touch i8, Maltron International Ltd, UK). All measurements were performed within the first 48 h of admission.

#### Handgrip strength

Handgrip strength was measured within the first 48 h of admission using an electronic hand dynamometer (Saehan Corporation, South Korea). All measurements were performed seated with shoulder and wrist in a neutral position and elbow flexed to 90°. Handgrip strength was based on the best attempt out of three with the dominant hand.

### Statistical analysis

Data were examined for normal distribution by histograms, quantile-quantile plots, and the Shapiro-Wilk test, while homogeneity of variance was explored by Levene’s Test. Missing observations were assumed to be missing completely at random. Parametric continuous variables were expressed as means with standard deviation (SD), and statistical comparisons were made with one-way analysis of variance (ANOVA), followed by Tukey’s post hoc test. Where normality and variance violations prohibited parametric analysis of data, non-parametric continuous variables were expressed as medians with interquartile range (IQR), and statistical comparisons between groups were made by Kruskal-Wallis one-way ANOVA on ranks, followed by Dunn’s post hoc test. Categorical variables were expressed as number (%), and group differences were tested with the Chi-squared (χ^2^) test. If differences were found, Bonferroni-corrected *p* values were obtained as appropriate to identify significant differences between groups. Prior to statistical analysis, all cytokine values were log-transformed. After log-transformation, data were considered normally distributed, and ANOVA was used to detect differences between groups, followed by Tukey’s post hoc test. After statistical analysis, cytokine data were back-transformed and expressed as geometric means with a 95% confidence interval (CI). Risk ratio (RR) with the corresponding 95% CI was calculated for the variables; ICU admission and mortality. All *p* values were two-sided, and the significance level was set at *p* < 0.05. All statistical analyses were performed using IBM SPSS Statistics 25.0 or GraphPad Prism 8.0.2.

### Ethical considerations

The study was approved by the scientific Ethics Committee at the Capital Region of Denmark (H-18024256), registered on ClinicalTrials.gov (NCT03795662), and conducted in accordance with the Declaration of Helsinki. Oral and written informed consent was obtained from all patients before enrollment.

## Results

**Fig. 1:**
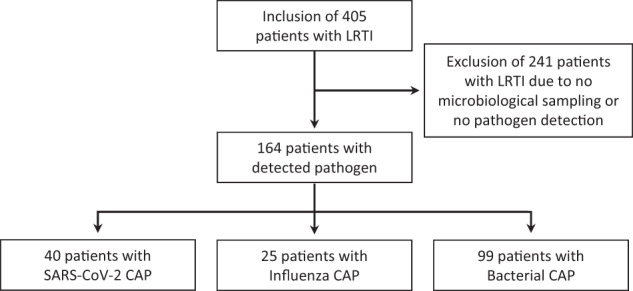
Flowchart showing the flow of the study population. *LRTI* lower respiratory tract infection.

Among 405 patients screened for inclusion, 164 (40.5%) fulfilled the criteria and were included in the study (Fig. 1). SARS-CoV-2 (*n* = 40, 24.4%) was the most common virus followed by influenza A (*n* = 25, 15.2%). In patients with bacterial CAP (*n* = 99, 60.4%), *Haemophilus influenzae* (*n* = 30, 30.3%) and *Streptococcus pneumoniae* (*n* = 18, 18.2%) were the two most common pathogens (Supplementary Table 1).

### Demography, comorbidities, and clinical parameters

Patient characteristics are summarized in Table 1. The groups were comparable in age, sex, CCI score, and the number of patients living in nursing homes. Patients with COVID-19 had fewer comorbidities (Table 1 and Supplementary Table 2). Regarding diabetes, 15.2% of the patients had known diabetes, 6.1% had undiagnosed diabetes, and 51.3% had pre-diabetes, but no difference between groups. According to the CURB-65 score, 57% of the patients had mild CAP, while 34% had moderate CAP, with no difference between groups. During hospitalization, 6 patients (15%) with COVID-19, 1 patient (4%) with influenza, and 4 patients (4%) with bacterial CAP were treated in the ICU, with a tendency towards a higher ICU admission rate among patients with COVID-19. Patients with COVID-19 had a higher risk of admission to the ICU than patients with influenza (RR 3.75, 95% CI 0.48–29.34) and bacterial CAP (RR 3.71, 95% CI 1.11–12.46). In addition, patients with COVID-19 had a tendency (*p* = 0.08) towards a higher in-hospital mortality risk compared to patients with influenza (RR 3.13, 95% CI 0.39–25.22) and bacterial CAP (RR 4.13, 95% CI 1.03–16.45). Furthermore, 30-days after discharge, patients with COVID-19 tended (*p* = 0.08) to have a higher mortality risk than patients with influenza (RR 3.13, 95% CI 0.39–25.22) and bacterial CAP (RR 4.13, 95% CI 1.03–16.45), respectively. At 180-days after discharge, the risk of mortality was still higher in patients with COVID-19 compared to patients with influenza (RR 1.88, 95% CI 0.41–8.58) and bacterial CAP (RR 1.86, 95% CI 0.69–5.01). The median length of hospital stay was 5 days (IQR: 3–10) for patients with COVID-19, which was similar to patients with influenza (5, IQR: 2–6) and bacterial CAP (5, IQR: 4–8; Table 1).

**Table 1:** Baseline characteristics.

	COVID-19 (*n* = 40)	Influenza (*n* = 25)	Bacterial (*n* = 99)
Demographic data			
Age, median (IQR), yrs.	72 (59–77)	72 (65–79)	73 (61–83)
Sex, male, *n* (%)	24 (60)	11 (44)	52 (53)
Nursing home resident, *n* (%)	2 (5)	2 (8)	5 (5)
Current smoker, *n* (%)	1 (3)	5 (23)*	17 (20)*
Comorbidities			
CCI, median (IQR)	3 (1–4)	4 (3–5)	4 (2–6)
Number of comorbidities			
0, *n* (%)	11 (28)	3 (12)	19 (19)
1, *n* (%)	19 (48)	5 (20)*	22 (22)**
≥2, *n* (%)	10 (25)	17 (68)***	60 (59)***
Known diabetes, *n* (%)	5 (12.5)	6 (24.0)	14 (14.1)
Undiagnosed diabetes, *n* (%)	3 (10.7)	1 (6.3)	3 (4.2)
Pre-diabetes, *n* (%)	17 (60.7)	7 (43.8)	35 (49.3)
Vital parameters on admission			
Systolic BP, median (IQR), mmHg	131 (116–150)	130 (121–156)	134 (118–149)
Diastolic BP, mean (SD), mmHg	72 (65–84)	73 (69–80)	74 (66–82)
Heart rate, mean (SD), beats/min	87 (16)	101 (22)**	99 (17)**
Respiratory rate, median (IQR) breaths/min	20 (19–22)	20 (18–24)	22 (19–25)
Temperature, median (IQR), °C	37.4 (36.9–38.1)	38.3 (37.4–38.7)	38.0 (37.3–38.6)*
Supplemental oxygen at admission, *n* (%)	9 (23)	16 (64)**	50 (51)**
CURB-65			
0–1, *n* (%), score	24 (60)	19 (76)	51 (52)
2, *n* (%), score	12 (30)	5 (20)	39 (39)
3–5, *n* (%), score	4 (10)	1 (4)	9 (9)
Disease outcome			
ICU admission, *n* (%)	6 (15)	1 (4)	4 (4)
Length of stay, median (IQR), days	5 (3–10)	5 (2–6)	5 (4–8)
In-hospital mortality, *n* (%)	5 (13)	1 (4)	3 (3)
30-day mortality, *n* (%)	5 (13)	1 (4)	3 (3)
180-day mortality, *n* (%)	6 (15)	2 (8)	8 (8)

One patient with bacterial CAP had a missing value on heart rate, and two patients with bacterial CAP had missing values on temperature. Comparisons were made with one-way ANOVA with Tukey post hoc test, Kruskal Wallis test with Dunn’s post hoc test, or χ^2^.

*BP* blood pressure, *CCI* Charlson comorbidity index, *CURB-65* score for pneumonia severity.

*P < 0.05; **P < 0.01; ***P < 0.001: Different from COVID-19.

### Body composition

Bodyweight, BMI, total FFM, FM, and FM percentage were similar between groups. In total, 60% of the patients had an increased FM percentage (males > 25% and females > 36%, respectively), with no difference between groups. Moreover, 33% of the patients were characterized with overweight, while 22% were characterized with obesity, with no difference between groups. In total, 82% of the female patients and 65% of the males had a higher waist circumference compared to the recommendation (>80 cm and >94 cm, respectively), with no difference between groups (Table 2). Thus, although most patients with COVID-19 had increased waist circumferences compared to the recommendation, the waist circumference was lower than in patients with influenza (Table 2).

**Table 2:** Physical capacity, body composition, and mental status.

	COVID-19 (*n* = 40)	Influenza (*n* = 25)	Bacterial (*n* = 99)
Physical capacity			
Handgrip strength, median (IQR), kg	23.2 (15.9–38.1)	23.0 (18.0–26.2)	23.3 (15.8–29.1)
Low PA level, *n* (%)	21 (84.0)	18 (94.7)	43 (79.6)
Moderate-to-high PA level, *n* (%)	3 (12.0)	1 (5.3)	11 (20.4)
Barthel Index-100, median (IQR), score	100 (100–100)	100 (90–100)	100 (90–100)
Body composition			
Bodyweight, median (IQR), kg	76.1 (70.6–88.6)	80.2 (64.9–93.8)	72.9 (58.8–87.6)
BMI, median (IQR), kg/m^2^	26.5 (23.1–29.2)	27.3 (23.9–31.6)	24.8 (21.7–28.6)
Waist, mean (SD), cm	96.6 (10.1)	110.4 (20.2)*	95.7 (15.9)
FFM, mean (SD), kg	55.1 (13.7)	51.7 (11.4)	50.0 (13.3)
FM, mean (SD), %	27.7 (8.0)	35.3 (10.5)	30.5 (10.5)
Mental status			
Quality of life, median (IQR), score	0.755 (0.638–0.826)	0.607 (0.406–0.692)*	0.680 (0.534–0.817)
Frailty, *n* (%)	2 (8)	4 (22)	14 (26)
Nutritional risk, *n* (%)	11 (44)	12 (63)	28 (50)

Seventy patients (SARS-CoV-2: 19, influenza: 5, bacterial: 46) had missing values on handgrip strength, 67 patients (SARS-CoV-2: 16, influenza: 6, bacterial: 45) had missing values on IPAQ score, and 63 patients (SARS-CoV: 10, influenza: 8, bacterial: 45) had missing values on Barthel Index-100. Forty-seven patients (SARS-CoV-2: 7, influenza: 5, bacterial: 35) had missing values on BMI, 89 patients (SARS-CoV-2: 20, influenza: 8, bacterial: 61) had missing values on waist circumference, and 86 patients (SARS-CoV-2: 30, influenza: 6, bacterial: 17) had missing values from bioelectrical impedance analysis scan. Fifty-seven patients (SARS-CoV-2: 11, influenza: 5, bacterial: 41) had missing values on quality of life, 64 patients (SARS-CoV-2: 11, influenza: 7, bacterial: 46) had missing values on frail scale, and 59 patients (SARS-CoV-2: 10, influenza: 6, bacterial: 43) had missing values on nutritional risk. Comparisons were made with one-way ANOVA with Tukey post hoc test, Kruskal Wallis test with Dunn’s post hoc test, or χ^2^.

*BMI* body mass index, *FFM* fat-free mass, *FM* fat mass, *PA* physical activity.

*P < 0.05: Different from COVID-19.

### Physical capacity and mental status

Overall, 83% of the patients had low handgrip strength, while 85% had low physical activity levels, with no difference between groups. The ability to perform daily activities was similar between groups. Moreover, frailty was seen in 22% of the patients, while 51% were at nutritional risk, with no difference between groups. In addition, patients with COVID-19 had a higher pre-hospital health-related quality of life than patients with influenza (Table 2).

### Metabolic and inflammatory parameters

Plasma adiponectin concentration, admission blood glucose, HbA1c, and lipids (LDL-cholesterol, HDL-cholesterol, total cholesterol, and triglycerides) were similar between the groups. Compared to normal values, 81% of the patients had admission hyperglycemia, while 60% had HDL-cholesterol <1.0 mmol/L, with no difference between groups (Table 3). Other laboratory parameters are shown in Supplementary Table 3.

**Table 3:** Glucose and lipid metabolism.

	COVID-19 (*n* = 40)	Influenza (*n* = 25)	Bacterial (*n* = 99)
Glucose, median (IQR), mmol/L	7.2 (6.6–8.1)	7.1 (6.1–8.2)	7.3 (6.3–8.5)
Glucose <5.99 mmol/L, *n* (%)	7 (18.4)	6 (24.0)	17 (17.3)
Glucose 6.0–10.99 mmol/L, *n* (%)	27 (71.1)	18 (72.0)	72 (73.5)
Glucose ≥11.0 mmol/L, *n* (%)	4 (10.5)	1 (4.0)	9 (9.2)
HbA1c (IFCC), median (IQR), mmol/mol	40 (38–46)	40 (37–50)	40 (36–44)
HbA1c 39–47 mmol/mol, *n* (%)	17 (51.5)	7 (31.8)	35 (41.7)
HbA1c ≥ 48 mmol/mol, *n* (%)	7 (21.2)	7 (31.8)	14 (16.7)
Plasma adiponectin, median (IQR), ×10^6 ^pg/ml	18.21 (14.22–24.97)	13.43 (9.88–19.58)	15. 76 (10.87–25.87)
LDL cholesterol, median (IQR), mmol/L	1.5 (1.1–1.9)	1.5 (1.0–2.2)	1.6 (1.2–2.3)
LDL cholesterol >3.00 mmol/L, *n* (%)	1 (3.3)	2 (9.5)	4 (4.9)
HDL cholesterol, median (IQR), mmol/L	0.85 (0.66–1.04)	0.97 (0.84–1.15)	0.88 (0.54–1.23)
HDL cholesterol <1.00 mmol/L, *n* (%)	22 (73.3)	11 (52.4)	47 (57.3)
Total cholesterol, median (IQR), mmol/L	3.1 (2.7–3.5)	3.2 (2.2–3.8)	3.2 (2.6–4.0)
Triglycerides, median (IQR), mmol/L	1.33 (1.11–1.65)	1.45 (1.10–1.89)	1.28 (0.94–1.44)

Three patients (SARS-CoV-2: 1, bacterial: 2) had missing values on blood glucose, 25 patients (SARS-CoV-2: 7, influenza: 3, bacterial: 15) had missing values on HbA1c, and 31 patients (SARS-CoV-2: 10, influenza: 4, bacterial: 17) had missing values on lipids. Comparisons were made with Kruskal Wallis test with Dunn’s post hoc test or χ^2^.

*HbA1c* hemoglobin A1c, *HDL* high-density lipoprotein, *IFCC* International Federation of Clinical Chemistry, *LDL* low-density lipoprotein.

### Metabolic and inflammatory parameters

**Fig. 2:**
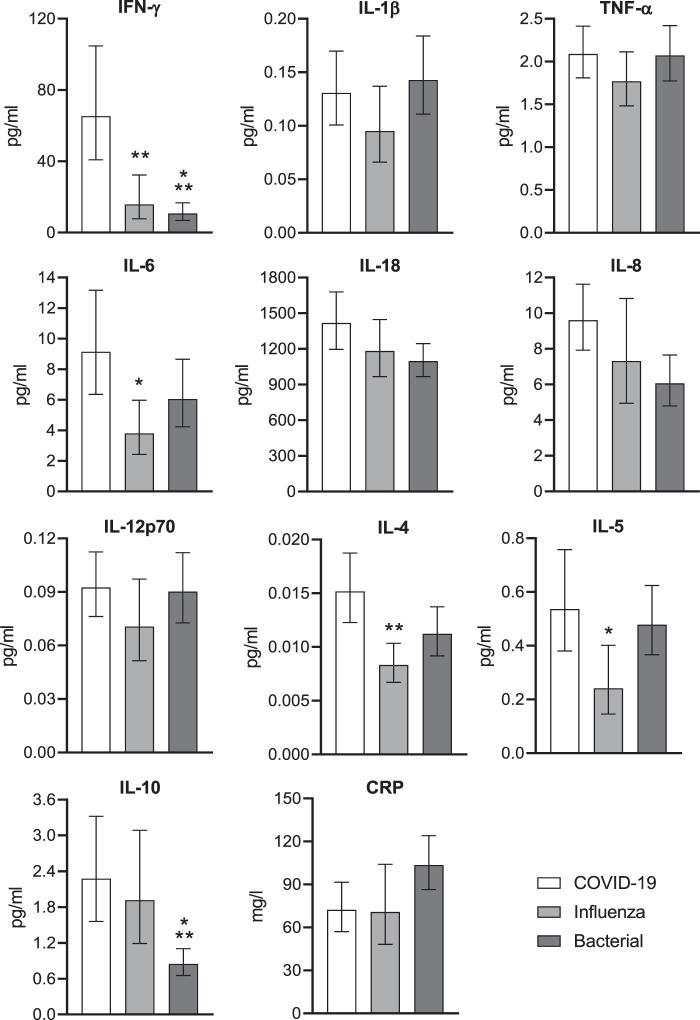
Cytokine concentration in plasma measured at admission. COVID-19 (*n* = 31): white bares, influenza (*n* = 24): bars in light gray, and bacterial CAP (*n* = 72): bars in dark gray. Data are in geometric means with 95% CI. Thirty-seven patients (SARS-CoV-2: 9, influenza: 1, bacterial: 27) had missing values on cytokine concentration, and four patients (SARS-CoV-2: 1, influenza: 1, bacterial: 2) had missing values on CRP concentration. Comparisons were made with one-way ANOVA with Tukey post hoc test. *P < 0.05; **P < 0.01, ***P < 0.001: Different from COVID-19.

Patients with COVID-19 had lower leukocyte count compared to patients with influenza and bacterial CAP and lower neutrophil and monocyte counts compared to patients with bacterial CAP (Supplementary Table 3). In addition, patients with COVID-19 had higher IFN-γ, IL-4, IL-5, and IL-6 concentrations compared to patients with influenza and higher IFN-γ and IL-10 concentrations compared to patients with bacterial CAP (Fig. 2).

## Discussion

Among Danish patients hospitalized with CAP, we found little difference in pre-admission status and the clinical presentation of CAP between different causative pathogens, including SARS-CoV-2, bacteria, and influenza virus. Overall, patients were characterized with a very high proportion of abdominal obesity, pre-diabetes, known and undiagnosed diabetes, and low HDL-cholesterol, but no difference between the groups. Likewise, most patients had low physical activity levels and handgrip strength, with no difference between the groups. Despite a similar pre-admission profile, the pro-and anti-inflammatory responses were more enhanced in patients with COVID-19 compared to patients with influenza and bacterial CAP.

The most common comorbidities among patients with COVID-19 were hypertension, asthma, and diabetes, which is partially comparable to a Danish nationwide study [24]. In comparison, similar comorbidities, including chronic obstructive pulmonary disease, were frequently seen in patients with influenza and bacterial CAP. A high CCI index (>2) has previously been associated with an increased risk of mortality from both COVID-19, influenza, and bacterial CAP [5, 16, 24]. In line with previous studies [18], pre-diabetes is common in patients with CAP, which we also found irrespective of the CAP etiology. In comparison, a recent study from the US of patients with moderate to severe COVID-19 reported 24% of the patients having pre-diabetes [25], a significantly lower number compared to our study. We have previously shown that among patients without a history of diabetes, pre-diabetes and undiagnosed diabetes were seen in 37.5 and 5% of the patients with bacterial CAP [18]. This high degree of pre-diabetes has not previously been shown in patients with COVID-19 but adds to the knowledge of an unhealthy metabolic phenotype in patients hospitalized with COVID-19. However, more evidence is needed to comment on the prognostic impact of pre-diabetes in the disease severity of COVID-19. However, patients with COVID-19 were more often admitted to the ICU and had higher mortality compared to patients with influenza and bacterial CAP.

People with obesity are predisposed to an increased susceptibility to CAP and increased risk of severe COVID-19 or influenza infection [6, 11, 26]. Interestingly, using bioelectrical impedance analysis, we found that the majority (60%) of the patients had an increased FM percentage (males > 25% and females > 36%, respectively), irrespectively of the CAP etiology, indicating general obesity in all three groups [27]. Furthermore, the adiposity was characterized by abdominal obesity as 73% of the patients had a waist circumference above the recommended [28]. In line with previous studies [6, 26], more than half of the patients were characterized with overweight or obesity, again with no difference between groups. A newly published retrospective French study comparing patients with COVID-19 and influenza [5] showed a higher proportion of patients with COVID-19 being characterized with overweight or obesity compared to patients with influenza. In contrast, overweight and obesity were more pronounced in our study independent of etiology compared to the previous study [5]. Differences in study design and/or patient population between the studies might explain the marked difference in the percentage of patients with COVID-19 and influenza being characterized with overweight or obesity. We included adult patients where the primary contact to the hospital was CAP, defined by new pulmonary infiltrate on chest x-ray/CT. In contrast, the French study [5] included patients where COVID-19 or influenza was either the primary or secondary diagnosis leading to hospitalization, indicating less severe infections in the French study.

Previous studies have, regardless of CAP etiology, demonstrated hyperglycemia at admission in hospitalized patients with CAP both with and without diabetes [29–31]. We add to the previous knowledge that no difference in hyperglycemia was observed between CAP etiology. Admission hyperglycemia in patients without diabetes has previously been associated with increased risk of critical illness and mortality from COVID-19 and bacterial CAP, possibly due to an impaired innate immunity [29–31]. Furthermore, in line with previous studies [32, 33], we observed that the majority (60%) of the patients had reduced HDL-cholesterol (<1 mmol/L) with no difference between groups. Dyslipidemia (high LDL-cholesterol and triglycerides and low HDL-cholesterol) is a risk factor for severe outcomes of COVID-19 and bacterial infections [32].

During pulmonary infections, IFN-γ plays a crucial role in host defense and regulation of inflammatory response [34]. There are essential differences between COVID-19 and influenza regarding the role of IFN-γ in driving disease severity. Previous studies have shown that reduced IFN-γ signaling in response to influenza improves clinically relevant outcomes by promoting the survival of CD8^+^ T cells [35]. This protective effect of low IFN-γ in influenza differs from COVID-19, where inadequate secretion of IFN-γ by CD4^+^ T cells correlates with disease severity [36]. Moreover, COVID-19 has been shown to induce a more robust IFN-γ response in vitro than influenza, suggesting that IFN-γ might play a larger part in the host immune response to COVID-19 [14]. This is supported by the present study, showing elevated IFN-γ levels in patients with COVID-19 compared to patients with influenza and bacterial CAP. However, the role of IFN-γ in the pathogenesis of bacterial CAP is conflicting and highly dependent on the type of bacteria [37]. Overall, high levels of TNF-α, IL-6, IL-8, and IL-10 have been associated with disease severity and poor survival from COVID-19 [15, 36, 38]. In critically ill patients with influenza, disease severity is also related to elevated levels of TNF-α, IL-6, IL-8, and IL-10 [39, 40]. As reviewed by Ritter and colleagues [12], people with obesity may be more susceptible to severe complications from the inflammatory response induced by the COVID-19 infection as a consequence of chronic low-grade inflammation and dysfunctional immune response. Current evidence could indicate that the obesity-induced inflammatory responses may be an essential driver of COVID-19 severity [41]. Given the same degree of general obesity and even lower degree of abdominal obesity in patients with COVID-19 compared to patients with influenza and bacterial CAP in our study, this could suggest that the severity of the interaction between obesity and systemic inflammation is enhanced in COVID-19. As patients with COVID-19 also had the lowest proportion of abdominal adiposity, this indicates that the increased inflammatory response observed in COVID-19 is not only explained by abdominal obesity nor dysregulated metabolic profile.

However, conclusive clinical data are still awaited as another study has not found any association between cytokine concentration and BMI in patients with COVID-19 [15]. Nevertheless, in the present study, IFN-γ, IL-4, IL-5, IL-6, and IL-10 were the only cytokines elevated in patients with COVID-19. In support, IL-4, IL-5, and IL-10 have previously been shown to increase over the course of disease in patients with severe COVID-19 [7, 38], suggesting an extensive upregulation of the adaptive immunity during COVID-19 [13].

### Study strengths and limitations

The study is a prospective cohort study, so real-time evaluation of outcomes such as body composition, functional capacity, and physical activity level between different CAP etiologies was possible. We used self-reported data, which can introduce information bias and misclassification. However, to reduce bias when collecting research, staff was trained by the same researcher. We cannot rule out the presence of selection bias in who was included in the study, as informed consent had to be obtained within the first 24 h of admission (consent bias). However, similar bias has occurred within each group and therefore has not affected the differences between the groups. As the study was a single-center study, geographic differences influencing the studied outcomes have been minimized. However, as with prospective studies, the patients were recruited at different time points. Patients with COVID-19 were recruited over two months, from March 24, 2020, to May 26, 2020. In contrast, patients with bacterial CAP were recruited over one and a half years from January 9, 2019, to May 25, 2020, while patients with influenza were recruited during seasonal influenza of 2018/2019 and 2019/2020. Finally, there was an imbalance in the number of patients in each group. The group with influenza CAP was relatively small compared to the other two groups.

## Conclusion

Despite similar metabolic phenotype and less abdominal adiposity, patients with COVID-19 demonstrated an increased inflammatory response, indicating that a dysregulated metabolic profile or abdominal obesity in patients with COVID-19 cannot be the only explanations for the more severe outcome seen in patients with COVID-19 compared to patients with other etiologies of CAP.

## Supplementary information

Supplementary material

## Data Availability

Relevant pseudonymized data will be available for other researchers on reasonable written request to the corresponding author.
