# Supplementary material for: Body composition, physical capacity, and immuno-metabolic profile in community-acquired pneumonia caused by COVID-19, influenza, and bacteria: a prospective cohort study
Source: Int J Obes (Lond). 2022 Jan 5;46(4):817–24. doi: 10.1038/s41366-021-01057-0 (PMC8729099; doi:10.1038/s41366-021-01057-0)
Supplement: Supplementary file 1 — Supplementary material [file 41366_2021_1057_MOESM1_ESM.pdf]

# Supplementary material

## Table 1. Etiology

| Pathogen | Study population (n = 164) | COVID-19 (n = 40) | Influenza (n = 25) | Bacterial (n = 99) |
|---|---|---|---|---|
| Virus, n (%) | | | | |
| SARS-CoV-2 | 40 (24.4) | 40 (100.0) | 0 (0.0) | 0 (0.0) |
| Influenza A | 25 (15.2) | 0 (0.0) | 25 (100.0) | 0 (0.0) |
| Parainfluenza | 1 (0.6) | 0 (0.0) | 0 (0.0) | 1 (1.0) |
| Bacteria, n (%) | | | | |
| *Haemophilus influenzae* | 31 (18.9) | 1 (2.5) | 0 (0.0) | 30 (30.3) |
| *Streptococcus pneumoniae* | 18 (11.0) | 0 (0.0) | 0 (0.0) | 18 (18.2) |
| *Escherichia coli* | 10 (6.1) | 1 (2.5) | 0 (0.0) | 9 (9.1) |
| *Staphylococcus aureus* | 7 (4.3) | 0 (0.0) | 0 (0.0) | 7 (7.1) |
| *Mycoplasma pneumoniae* | 7 (4.3) | 0 (0.0) | 0 (0.0) | 7 (7.1) |
| *Legionella pneumophila* | 5 (3.0) | 0 (0.0) | 0 (0.0) | 5 (5.1) |
| *Moraxella catarrhalis* | 5 (3.0) | 1 (2.5) | 0 (0.0) | 4 (4.0) |
| *Pseudomonas aeruginosa* | 4 (2.4) | 0 (0.0) | 0 (0.0) | 4 (4.0) |
| Others | 49 (29.9) | 4 (10.0) | 0 (0.0) | 45 (45.5) |

## Table 2. Comorbidities

| Comorbidities, n (%) | COVID-19 (n = 40) | Influenza (n = 25) | Bacterial (n = 99) |
|---|---|---|---|
| Hypertension | 18 (45) | 15 (60) | 42 (42) |
| Asthma | 5 (13) | 5 (20) | 18 (18) |
| Diabetes | 5 (13) | 6 (24) | 14 (14) |
| Malignancy | 4 (10) | 2 (8) | 15 (15) |
| Cerebrovascular disease | 4 (10) | 5 (20) | 14 (14) |
| COPD | 3 (8) | 10 (40) | 35 (35) |
| Heart failure | 1 (3) | 3 (12) | 12 (12) |
| Other neurological disease | 2 (5) | 2 (8) | 4 (4) |
| Chronic liver disease | 0 (0) | 1 (4) | 3 (3) |
| Chronic kidney disease | 0 (0) | 1 (4) | 2 (2) |

## Table 3. Laboratory results

| | COVID-19 (n = 40) | Influenza (n = 25) | Bacterial (n = 99) |
|---|---|---|---|
| Leucocytes, median (IQR), ×$10^9$ cells/L | 6.2 (4.8-8.3) | 8.6 (5.9-11.2)* | 10.8 (8.6-13.7)*** |
| Lymphocytes, median (IQR), ×$10^9$ cells/L | 0.90 (0.80-1.40) | 1.35 (0.90-1.83) | 1.20 (0.80-1.80) |
| Monocytes, median (IQR), ×$10^9$ cells/L | 0.4 (0.3-0.7) | 0.7 (0.4-0.9) | 0.8 (0.5-1.0)*** |
| Neutrophils, median (IQR), ×$10^9$ cells/L | 4.6 (3.1-5.9) | 6.2 (3.9-9.3) | 8.5 (6.2-10.3)*** |
| Thrombocytes, median (IQR), ×$10^9$ cells/L | 235 (171-305) | 189 (180-299) | 261 (192-341) |
| Hemoglobin, mean (SD), mmol/L | 8.0 (0.9) | 7.7 (1.0) | 7.4 (1.0)** |
| Ferritin, median (IQR), µg/L | 775 (440-1460) | 305 (245-380)*** | 322 (191-553)*** |
| Albumin, mean (SD), g/L | 26 (4) | 28 (1) | 26 (5) |
| Creatinine, median (IQR), µmol/L | 76 (62-87) | 70 (57-87) | 74 (58-86) |
| Urea, median (IQR), mmol/L | 5.6 (3.7-7.8) | 6.3 (5.1-8.0) | 7.1 (4.9-10.6) |
| D-dimer, median (IQR), FEU/L | 1.4 (1.1-1.6) | 2.2 (1.0-3.3) | 1.7 (0.7-2.6) |
| INR, median (IQR) | 1.0 (1.0-1.1) | 1.1 (1.0-1.1) | 1.1 (1.0-1.1) |
| ALAT, median (IQR), U/L | 26 (18-37) | 23 (19-39) | 23 (13-39) |
| Bilirubin, median (IQR), µmol/L | 10 (9-12) | 7 (5-10)** | 8 (6-13) |

| | | | |
|---|---|---|---|
| LDH, median (IQR), U/L | 276 (230-344) | 245 (198-297) | 189 (164-219)*** |
| Procalcitonin, median (IQR), µg/L | 0.09 (0.07-0.25) | 0.11 (0.08-0.24) | 0.31 (0.10-3.84)** |

Note: ALAT: Alanine aminotransferase, INR: International normalized ratio, LDH: Lactate dehydrogenase. *$P$<0.05; **$P$<0.01, ***$P$<0.001: different from COVID-19. Comparisons were done by Kruskal Wallis Test with Dunn's post hoc test or one-way ANOVA with Tukey post hoc test.
